# Les complications chirurgicales de la transplantation rénale à partir du donneur vivant: expérience du CHU Ibn Sina de Rabat

**Published:** 2010-09-18

**Authors:** Haddiya Intissar, Skalli Zoubeir, Benamar Loubna, Fatima Ezzaitouni, Ouzeddoun Naima, Bayahia Rabia, Rhou Hakima

**Affiliations:** 1 Nephrology- Dialysis-Renal transplantation Department, Ibn Sina University Hospital, Rabat- Morocco

**Keywords:** Complications chirurgicales, Lymphocèle, Sténose artère, Sténose urétérale Thrombose vasculaire, Transplantation rénale, Maroc

## Abstract

**Introduction:**

La transplantation rénale (TR) est actuellement considérée comme un traitement de choix de l’insuffisance rénale chronique terminale (IRCT). Ses résultats se sont améliorés au cours des dernières années. Cependant, les complications chirurgicales demeurent graves car elles touchent un rein unique et surviennent sur un terrain fragilisé par l’insuffisance rénale et l’immunosuppression. L’objectif de ce travail est d’évaluer la fréquence des complications chirurgicales lors de l’activité de TR au CHU Ibn Sina de Rabat, et de dégager les facteurs ayant influé l’apparition de ces complications.

**Méthodes:**

Étude rétrospective des patients transplantés rénaux à partir de donneurs vivants apparentés (DVA) de Juin 1999 à Décembre 2008 dans notre centre hospitalo-universitaire. Nous avons recensé les caractéristiques propres au receveur, au prélèvement, au donneur ainsi qu’au greffon. Les complications chirurgicales ont été colligées ainsi que leur prise en charge et évolution.

**Résultats:**

Soixante sept dossiers ont été analysés avec un suivi moyen de 55 +/- 28 mois. 38 complications chirurgicales ont été recensées : sténose des artères rénales (38,7%), lymphocèle (21%), hématome (12,7%), thrombose vasculaire (7,8%), reflux  ésico-urétéral (4,8%), rupture du greffon (3,2%), calcul (1 cas), éventration (1 cas), L’analyse statistique de notre série n’a pas mis en évidence de facteurs de risque significatifs semblant influer sur l’incidence des complications chirurgicales.

**Conclusion:**

La morbidité liée aux complications chirurgicales de la TR reste élevée nécessitant un diagnostic et un traitement adéquat afin d’éviter les répercussions sur la survie des patients et des greffons.

## Introduction

En Décembre 1954, Murray, Merrill et Harrisson réalisaient la première TR entre deux vrais jumeaux. Aujourd’hui, une cinquante d’années plus tard, la TR est considérée comme le traitement de choix de l’IRCT sur le plan médical et économique. Ses résultats se sont améliorés au fil du temps avec une nette augmentation de la durée de vie des transplants et une baisse des complications tant médicales que chirurgicales. Le rejet, autrefois redouté à cause de sa fréquence élevée et de sa gravité est devenu moins fréquent [1]. Quant aux complications chirurgicales, elles ont diminué en incidence et en gravité [2,3].

Cette amélioration des résultats est multifactorielle. Les techniques chirurgicales ont beaucoup évolué en ce qui concerne à la fois le le prélèvement et l’implantation. Toutefois, les complications chirurgicales restent possibles sur le plan urologique (4 à 8%) et vasculaire (1 à 2 %) [4]. Elles sont graves car elles touchent des patients fragilisés par l’insuffisance rénale et traitement immunosuppresseur et porteurs d’un rein fonctionnellement unique, d’où la nécessité de les reconnaître le plus tôt possible et de proposer le traitement le plus adéquat. Elles sont variées et souvent spécifiques de la transplantation.

L’objectif de cette étude est d’étudier les complications chirurgicales de la TR dans notre série, leur fréquence, les facteurs ayant influencé leur survenue ainsi que leur prise en charge, leur évolution et le retentissement à court et à long terme sur la fonction du greffon.

## Méthodes

Il s’agit d’une étude rétrospective incluant tous les patients ayant bénéficié d’une première TR par donneur vivant (DV) de Juin 1998 à Décembre 2008 au service de TR au CHU Ibn Sina de Rabat.

Les caractéristiques propres au receveur, au prélèvement, au donneur ainsi qu’au greffon (GR) ont été établies. Les complications chirurgicales ont été colligées.

Les caractéristiques suivantes ont été recensées: 1) Les caractéristiques propres aux receveurs: L’âge, le sexe, la néphropathie initiale, l’ancienneté de la dialyse, l’index de masse corporelle (IMC), un surpoids était considéré pour un IMC>25, 2) les caractéristiques du greffon, du prélèvement; et de l’implantation: Le type de DV, le rein prélevé. Une laparotomie droite est effectuée chez tous nos patients de telle sorte à placer le greffon rénal dans la fosse iliaque droite. L’artère rénale du greffon est anastomosée en termino-latérale sur l’artère iliaque externe. La veine rénale du greffon est anastomosée en termino-latérale sur la veine iliaque externe. L’anastomose urétéro-vésicale est de type Lich -Gregoir avec mise en place d’une sonde en double j enlevée au 21ème jour sauf si le patient fait une infection urinaire, dans ce cas elle est retirée plus tôt. La sonde vésicale est enlevée à J+5.

Tous les patients ont eu des contrôles systématiques par l’échographie du greffon rénal (GR) et le doppler des vaisseaux du greffon à J+1 et à J+5. Des contrôles plus fréquents étaient demandés devant des anomalies cliniques ou à l’imagerie. A la sortie de l’hôpital, tous les patients ont bénéficié de contrôles systématiques par échographie et doppler à 3 mois, 6 mois puis annuellement.

L’analyse statistique est réalisée à l’aide du logiciel SPSS 10. Les variables quantitatives sont exprimées en moyennes – écart types quand elles répondent à la loi normale et en médianes – interquartiles: 25-75 quand elles sont hors la loi normale. Les variables qualitatives sont exprimées en pourcentage. La comparaison des moyennes quantitatives est réalisée à l’aide du test t de Student et le test exact de Fisher et la comparaison des variables qualitatives à l’aide du test de Chi-carré.

## Résultats

Sur une période de 10 ans, nous avons colligés 67 patients ayant bénéficié d’une première TR par DVA dans le service de TR du CHU Ibn Sina de Rabat. L’âge moyen de nos patients est de 30 +/- 9,6 ans (13 - 56), avec 64,1% % des hommes et 35,8% des femmes. 14,2% des patients présentent un surpoids. La néphropathie initiale était indéterminée dans 59,7 % des cas.

Nos patients étaient traités par hémodialyse, avant la greffe, dans 65 cas (97%), et dialyse péritonéale dans 2 cas (2,9%). La durée de dialyse médiane est de 24 mois (2 – 260). Concernant le greffon et le prélèvement, la moyenne d’âge des donneurs est de 42,4 +/- 11,08 ans (20 – 61). Les donneurs âgés de plus de 50 ans représentaient 26,4% de l’ensemble des donneurs. Dans 47 cas (70,1%) les donneurs étaient de sexe féminin alors que dans 20 cas (29,8%) ils étaient de sexe masculin. Le donneur est la mère, la sœur, le frère, le père, le mari et le fils dans respectivement 28 cas (41,7%), 19 cas (28,3%), 3 cas (4,4%), 15 cas (22,3%), 1 cas (1,4%), 1cas (1,4%).

La néphrectomie a intéressée le rein gauche dans 60 cas (89,5%) cas avec une artère et une veine dans 60 cas (89,5%) des cas, 2 artères dans 6 cas (8,9%) et 3 artères dans un cas (1,4%). Sur un suivi moyen de 55+/- 28 mois, 38 complications chirurgicales ont été recensées et de gravité variable.

**A. Description des complications**

**Les complications urologiques**

Reflux vésico-urétéral (RVU)

Trois RVU (4,8%), faisant suite à des infections urinaires à répétition, ont été retrouvés à l’UCG avec un délai moyen d’apparition de 43+/- 37 mois (10 à 84 mois) après la TR. Le traitement était médical à base d’antibiothérapie adaptée et prolongée sans cure chirurgicale. L’évolution était bonne pour les trois patients avec un bon fonctionnement du greffon à court et à long terme.

Sténose urétérale

Deux sténoses urétérales (3,2%) ont été colligées dans la population des malades transplantés dans notre centre avec un délai d’apparition de 46 mois, un à 2 mois et le deuxième à 9 à mois après la TR. Elles ont été diagnostiquées à l’échographie et la scintigraphie rénale. Deux patients ont bénéficié d’une montée de sonde en double j. l’évolution était bonne pour les deux patients sans retentissement sur le greffon à court et à long terme.

Calcul

Un patient a présenté un calcul rénal avec une insuffisance rénale aigue obstructive et une dilatation pyélo-calicielle diagnostiquée à l’échographie rénale à 21 mois de la TR. Il a bénéficié d’une néphrostomie en urgence avec élimination spontanée du calcul par voie urinaire.

**Les complications vasculaires**

Thrombose vasculaire

Trois patients (4,7%) ont présenté une thrombose de l’artère du GR diagnostiquée au doppler des vaisseaux rénaux à J0 dans deux cas et j4 dans 1 cas. Deux patients ont bénéficié d’un traitement à base d’héparine relayé par les antivitamines K et des vasodilatateurs avec une bonne évolution dans 1 cas (créatinine actuelle à 29 mg/l avec un recul de 3 ans). Deux patients ont été détransplantés. Une patiente a présenté une thrombose veineuse diagnostiquée au doppler des vaisseaux rénaux à J0 de la TR. L’évolution a été marquée par une détransplantation.

Sténose des artères rénales

Vingt-quatre patients (38,7%) ont présenté une sténose de l’artère du greffon dans un délai moyen de 33 jours. 19 patients ont été traités médicalement, 5 ont bénéficié d’une angioplastie dont 3 avec mise en place d’un stent devant une HTA sévère avec aggravation de la fonction rénale. L’évolution était bonne avec récupération de la fonction rénale et un équilibre tensionnel après 3 ans de recul.

Rupture du greffon

Nous avons relevé 2 ruptures de greffon (3,2%) dans notre série, survenues respectivement le 4ème et 5ème jour de la TR, dans un tableau d’altération de la fonction rénale avec douleurs abdominales. La rupture du greffon était secondaire à une nécrose tubulaire aigue diagnostiquée à la biopsie rénale chez un patient et aucune étiologie n’a été retrouvée chez le deuxième. L’exploration, en urgence au bloc opératoire, retrouvait une rupture corticale polaire, avec un greffon satisfaisant pour le reste conduisant à une conservation après réparation et conditionnement du rein dans un filet de vicryl. Les 2 greffons sont toujours fonctionnels avec un recul de 5 et 6 ans respectivement.

Par ailleurs aucun de nos patients transplantés n’a présenté de fistule artério-veineuse ni pseudo-anévrysme. Ces derniers surviennent en général après biopsie du greffon.

hémorragies

Huit patients (12,7%) ont présenté un hématome sans retentissement hémodynamique avec un délai moyen d’apparition de 4 jours. Deux patients ont bénéficié d’une reprise chirurgicale avec évacuation de l’hématome et transfusion de culots globulaires. L’évolution était bonne pour tous les patients.

Lymphocèle

Treize lymphocèles (21%) ont été diagnostiqués dans le suivi des patients avec un délai médian de 6 mois (1+/- 20 mois). Trois ont nécessité une marsupialisation devant un lymphocèle compressif. On n’a pas noté de récidive après le geste chirurgical.

**Les complications pariétales**

Un patient a présenté une éventration après reprise chirurgicale pour détransplantation rénale.

**B. Facteurs pronostiques**

L’analyse statistique univariée croisant les paramètres définis dans la méthode, concernant le donneur, le receveur et la survenue de complications n’a pas mis en évidence de facteurs de risque significatifs dans notre série (Tableau 1, 2, 3). Cependant, la survenue d’une complication vasculaire est un facteur de risque significatif associé à la dégradation de la fonction du greffon rénal à 5 ans (p=0,011); alors que la survenue d’une complication urologique n’est pas significativement corrélée à la dégradation de la fonction du greffon rénal à 5 ans (p=0,313) (Tableau 4, 5, 6).

## Discussion

La fréquence des complications observées dans notre série est globalement comparable aux données de la littérature. En effet, nous avons rapporté un taux de 4,8% de RVU symptomatiques, découvert à l’occasion d’infections urinaires récidivantes, ou d’altération de la fonction rénale. L’incidence de cette complication dans la littérature est variable et peu connue. Certains auteurs rapportent un taux variant de 10 à 80% [3]. Dans notre série, le traitement était médical à base d’antibiothérapie adaptée et prolongée sans cure chirurgicale. L’évolution était bonne pour les trois patients avec un bon fonctionnement du greffon à court et à long terme.

La fréquence des sténoses urétérales (3,2%) dans notre série était conforme aux données de la littérature [5-7]. La cause principale est la sténose de l’uretère distal d’origine ischémique [7]. La majorité des sténoses surviennent dans l’année qui suit la TR, dont 70% dans les 3 premiers mois. La prévention est basée sur le respect de la vascularisation urétérale et la rigueur dans la réalisation de l’anastomose uretèro-vésicale.

La faible incidence des calculs chez les transplantés rénaux, de 0,3 à 1% contraste avec la fréquence des troubles métaboliques tels que l’hyperparathyroïdie. Ils peuvent être transmis par le transplant lui-même ou apparaître de novo [8]. Ils touchent plus souvent les femmes que les hommes contrairement à ce qui est observé dans la population générale. Le traitement repose sur La lithotritie extracorporelle qui a été utilisée depuis le début des années 1990 [9]. Elle donne d>'excellents résultats même si dans certains cas la néphrostomie est nécessaire pour éviter l'obstruction urétérale par les fragments lithiasiques [8]. La néphrolithotomie percutanée peut aussi être pratiquée [10,11]. L'urétéroscopie a également été utilisée [12,13]. Dans notre série un seul patient a présenté un calcul rénal avec une insuffisance rénale aigue obstructive avec une dilatation pyélo-calicielle diagnostiquée à l’échographie rénale à 21 mois de la TR. Il a bénéficié d’une néphrostomie en urgence avec élimination du calcul par voie urinaire.

Les complications chirurgicales d’ordre vasculaire peuvent toucher l’artère ou la veine du transplant. Elles sont devenues rares mais elles ont gardé leur gravité avec le plus souvent une perte du greffon. L’incidence des thromboses veineuses varie de 0,4% chez l’adulte à 2,5% chez l’enfant. Le diagnostic est souvent tardif en dehors de quelques cas anecdotiques de sauvetage, le transplant est perdu [14,15]. La détransplantation est le seul traitement possible pour la majorité des équipes. En dehors d’une malfaçon technique ou d’une veine particulièrement abîmée au moment du prélèvement, la cause reste mystérieuse. Il pourrait s’agir de problèmes hémodynamiques avec chutes fréquentes ou prolongées de la tension artérielle, voire de problèmes immunologiques (rejet aigu), mais aucune preuve n’a été fournie concernant en particulier cette dernière hypothèse. Par ailleurs, deux autres facteurs favorisants ont été évoqués: une hypercoagulabilité et la mutation du gène du facteur V Leiden. Le véritable traitement est donc préventif: allonger la veine rénale par un patch de veine cave pour les transplants droits, éviter toute plicature ou traction sur le pédicule vasculaire par un choix judicieux des sites d’anastomose vasculaire, prescrire un traitement anticoagulant adapté et maintenir un état hémodynamique stable.

La thrombose artérielle est encore plus rare, de 0,4% [24] à 2% [16]. Elle est souvent liée à des problèmes techniques : anastomose de mauvaise qualité, lésions d’athérome chez le receveur et le donneur, dissection de l’intima de l’artère du transplant passée inaperçue, dissection de l’artère iliaque apparue secondairement au niveau de la zone de clampage avec en plus des signes d’ischémie du membre inférieur homolatéral. Le traitement est avant tout préventif, car le pronostic est mauvais [17]. Cette complication requiert dans la majorité des cas la détransplantation: c’est le cas de 3 patients parmi les 4 de notre série concernés par cette complication.

La sténose des artères rénales (SAR) est une complication qui survient dans la majorité des cas, après le troisième mois et dans l’année qui suit la TR. Sa prévalence varie de 1 à 23% selon les séries [18-20]. Dans notre étude la SAR a été observée dans 38,7% des cas, avec un délai d’apparition de trois mois à deux ans. Cependant la plupart des de ces SAR sont minimes sans retentissement sur la fonction du greffon ni majoration de l’HTA, la SAR n’a été jugée significative que chez cinq patients (7,4%) d’où l’indication d’une revascularisation. La SAR s’explique par la généralisation des contrôles par écho-doppler permettant de découvrir des sténoses asymptomatiques. Le traitement est indiqué si la sténose est symptomatique ou si le risque de thrombose est élevé. L’angioplastie avec ou sans stent donne de bons résultats dans 70% des cas même si c’est parfois au prix d’une seconde dilatation. Les risques ne sont pas négligeables : échec de dilatation, dissection de l’intima avec risque de thrombose, hémorragie par rupture artérielle et enfin néphro-toxicité des produits iodés utilisés. La chirurgie donne aussi de bons résultats, mais avec une morbidité importante et un risque de perte du transplant plus élevés qu’avec le traitement percutané. Elle est actuellement réservée aux sténoses non accessibles à l’angioplastie.

Une autre complication chirurgicale grave en TR est la rupture du greffon, qui menace à la fois le greffon et le receveur. Elle était souvent associée à un rejet aigu et à, moindre fréquence, à une tubulopathie aigue sévère [21]. Elle est devenue exceptionnelle. Les seuls cas de sauvetage ont été rapportés chez des patients ayant plutôt une tubulopathie sévère qu’un rejet aigu [21]. Dans notre série, nous avons relevé 2(3,2%) ruptures du greffon, secondaire à une nécrose tubulaire aigue diagnostiquée à biopsie du greffon chez un patient, alors qu’aucune étiologie n’a été retrouvée chez le deuxième. L’exploration, en urgence, retrouvait une rupture corticale polaire, avec un greffon satisfaisant pour le reste conduisant à une conservation après réparation et conditionnement du rein dans un filet de Vicryl. Les 2 greffons sont toujours fonctionnels avec un recul de 5 et 6 ans respectivement.

Les complications hémorragiques quant à elles, peuvent survenir dans les suites immédiates, dés la fin de l’intervention. L’hémorragie provient le plus souvent d’un défaut d’hémostase au niveau du hile rénal ou au niveau de la loge crée pour placer le transplant. Elle peut aussi compliquer les biopsies percutanées et écho-guidées des transplants rénaux (2,5%) [22]. Plusieurs facteurs favorisants ont été rapportés: difficultés de dissection chez un receveur obèse, mauvaise préparation du transplant avec défaut de ligature au niveau de la graisse du hile rénal, existence de traitement antiagrégant plaquettaire, mise en route d'un traitement anticoagulant à des doses importantes en cas de prothèse valvulaire cardiaque par exemple. Les transplants avec artères multiples à partir de donneurs vivants comportent plus de risque d'hémorragie [17,23]. La prévention reste le meilleur traitement : lier tout ce qui est sectionné au niveau du hile rénal, effectuer une hémostase rigoureuse et contrôler quotidiennement l’hémostase en cas de traitement par héparine. Dans notre série huit patients (12,7%) ont présenté un hématome sans retentissement hémodynamique avec un délai moyen d’apparition de 4 jours. Deux ont bénéficié d’une reprise chirurgicale avec évacuation de l’hématome et transfusion de culots globulaires. L’évolution était bonne pour tous les malades.

Les lymphocèles ont une incidence qui varie de 1 à 20%. Cette variabilité est probablement en rapport avec la définition du lymphocèle dont la recherche n’était pas systématique. Une augmentation de l’incidence a été signalée récemment par les équilibres utilisant les inhibiteurs des récepteurs mTOR [24]. La prévention repose sur une ligature minutieuse au fil non résorbable des tissus au niveau du hile rénal, une dissection limitée des vaisseaux iliaques et l’utilisation de clips ou de ligatures non résorbables. Le traitement est justifié si la collection est volumineuse, douloureuse ou si elle comprime la voie excrétrice du transplant. La laparoscopie est actuellement le traitement de première intention. Elle permet la résection de la partie saillante de la lymphocèle et la création d’une large communication avec le péritoine pour éviter la récidive. Dans notre série, treize lymphocèles (21%) ont été diagnostiqués dans le suivi des patients avec un délai médian de 6 mois (1+/-20 mois). Trois ont nécessité une marsupialisation devant un lymphocèle compressif. On n’a pas noté de récidive après le geste chirurgical.

Dans notre série un patient a présenté une éventration après reprise chirurgicale pour détransplantation rénale. Cette complication s’observe dans 3 à 5% des cas. Elle est favorisée par les reprises chirurgicales, le diabète, l’obésité, le rejet, l’infection pariétale et enfin par les corticoïdes et les inhibiteurs du mTOR [25]. Le traitement est identique à celui proposé dans la population générale avec recours aux treillis synthétiques chaque fois que nécessaire.

## Conclusion

La fréquence des complications chirurgicales de la TR a nettement diminuée grâce à l’amélioration des techniques chirurgicales. Elles sont dominées par les problèmes de l’anastomose urinaire et ce malgré l’amélioration des techniques ces dernières années. Cependant, elle reste relativement bénigne puisqu’il n’y a pas de retentissement évident sur le greffon à long terme. Les complications vasculaires et en particulier les thromboses vasculaires, sont de mauvais pronostic et leur traitement est essentiellement préventif.

## Conflits d’intérêts

Les auteurs ne déclarent aucun conflit d’intérêts

## Contribution des auteurs

Tous es auteurs ont participés a l’étude conformément aux critères de l’ICMJE

## Figures and Tables

**Table 1:** Complications chirurgicales de la transplantation rénale

**Complication**	**Fréquence**	**Traitement**	**Evolution**
Hémorragie de paroi	1 cas (1,4%)	Reprise chirurgicale de paroi avec hémostase	Favorable
Eventration	1 cas (1,4%)	Chirurgie d’éventration	Favorable
lymphocèle	13 cas (19,4%)	Marsupialisation: (3 cas)	Favorable
Reflux vésico-urétéraux	3 cas (4,47%)	Antibiothérapie	Favorable
Sténose de l’uretère	1 cas (1,4%)	Montée de sonde JJ	Favorable
Rupture de greffon	2 cas (2,9%)	Treillis de vicryl	Favorable
Hématomes	6 cas (8,9%)	Evacuation chirurgicale 2 cas	Favorable
Sténose de l’artère rénale	24 cas (38,7%)	Angioplastie seule : 2 cas	
		Angioplastie avec stent : 3 cas	Re-sténose: 1 cas

**Tableau 2:** Lien de parenté des donneurs avec les transplantés

**Sexe**	**Lien de parenté**	**Nombre (%)**
**Femmes**	Mère	28(41,7%)
47 (70,1%)	Sģur	19 (28,3%)
**Hommes**	Père	3 (4,4%)
20 (29,8%)	Frère	15 (22,3%)
	Mari	1 (1,4%)
	Fils	1 (1,4%)

**Tableau 3:** Facteurs de risque associés à la survenue de complications chirurgicales

**Caractéristiques**	**Patients présentant une complication chirurgicale.**	**Patients sans complications chirurgicales.**	**P**
	**N=37 (55,2%)**	**N=30 (44,8%)**	
Age moyen des receveurs (ans)	35,3 ± 11,2	33,2 ± 8,2	0,6
Sex-ratio (Homme/Femme)	2,7 (27H/10F)	1,30 (17H/13F)	0,051
Durée médiane de dialyse (mois)	19,5 (5-260)	35 (2-96)	0,580
Age des donneurs (ans)	43,5 ± 10,3	39,4 ± 10,7	0,113

**Tableau 4:** Influence des complications vasculaires sur la fonction rénale (analyse univariée)

**Fonction du greffon rénal**	**Patients présentant une complication vasculaire.**	**Patients sans complications vasculaires.**	**P**
Créatininémie moyenne à 5 ans.	N= 36 (53%)	N=31 (47%)	0,011
(mg/l)	15,3 ± 4,2	11,7 ± 2,8	

**Tableau 5:** Influence des complications urologiques sur la fonction rénale (analyse univariée)

Fonction du greffon rénal	Patients présentant une complication urologique. N= 4 (5,9%)	Patients sans complications urologiques. N=63 (94,1%)	p
Créatininémie moyenne à 5 ans. (mg/l)	16,5 ± 3,5	13,3 ± 4,2	0,313

**Tableau 6:** Influence des complications vasculaires et urologiques sur la fonction rénale (analyse univariée)

**Complications chirurgicales**	**Patients présentant une créatininémie à 5 ans < 12 mg/l. N=46 (68,6%)**	**Patients présentant une créatininémie à 5 ans E 12 mg/l N= 21 (31,3%)**	**P**
Complications vasculaires	25 (54,3%)	11 (52,4%)	0,011
Complications urologiques	2 (4,3%)	2 (9,5%)	0,313
